# Sequential therapy with inotuzumab ozogamicin, CD19 CAR T cells, and blinatumomab in an elderly patient with relapsed acute lymphoblastic leukemia

**DOI:** 10.1007/s00277-020-04227-8

**Published:** 2020-08-22

**Authors:** Ramona Wullenkord, Christian Reicherts, Jan-Henrik Mikesch, Julia Marx, Klaus Wethmar, Jörn Albring, Simon Call, Georg Lenz, Matthias Stelljes

**Affiliations:** Department of Medicine A, Hematology and Oncology, University of Münster, Albert-Schweitzer-Campus 1, 48149 Münster, Germany

Dear Editor,

Whereas outcome in younger patients with acute B-lymphoblastic leukemia (B-ALL) improved over the last decades, prognosis for older patients, especially with relapsed or refractory (r/r) disease, remains particularly dismal [1, 2]. Novel targeted approaches including inotuzumab ozogamicin (InO), blinatumomab (Blina), and CD19-directed chimeric antigen receptor (CAR) T cells demonstrated promising efficacy in recent phase II/III trials [3–9]. Here, we report a case of an elderly patient with repeatedly relapsed ALL treated sequentially with InO, CD19-specific CAR T cells, and finally, with Blina for relapse after CAR T cell therapy.

**Fig. 1:**
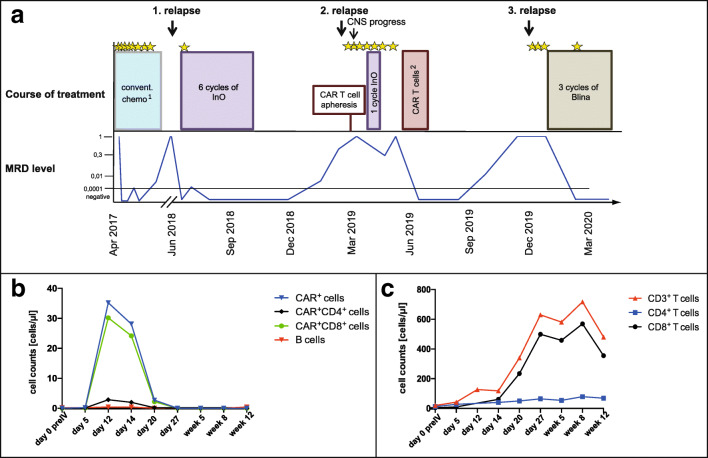
**a** Illustration of the course of treatment and MRD level over time. Asterisks indicate intrathecal administrations of chemotherapy. (1) Conventional chemotherapy according to the treatment recommendation for elderly patients of the GMALL study group. (2) CAR T cell administration after lymphodepleting chemotherapy with fludarabine and cyclophosphamide. **b** Persistence of CD19 CAR T cells in peripheral blood after CAR T cell administration (day 0) over time: the graphs depict the absolute cell counts of all circulating CD19 CAR T cells (blue curve) and the CAR^+^CD4^+^ (black curve) and the CAR^+^CD8^+^ T cell subsets (green curve). The red curve illustrates the absolute number of B cells. *preIV*, prior to intravenous CAR T cell infusion. **c** T cell counts after CAR T cell administration (day 0) over time: the graph illustrates the number of CD3^+^ T cells per microliter by the red curve, and of the CD4^+^ and CD8^+^ T cell subsets by the blue and black curves, respectively. Detection of B cells, T cells, and CD19 CAR T cells and their subsets was performed by flow cytometry (MACSQuant® Analyzer 10) using the following reagent and antibodies by Miltenyi Biotec: CD19-CAR detection reagent Biotin, anti-human Biotin-PE antibody (clone REA746), anti-human CD4-VioGreen™-antibody (clone REA623), anti-human CD3-FITC-antibody (clone REA613), anti-human CD19-PE antibody (clone REA675), and anti-human CD8-APC-Vio®770 antibody (clone REA734)

In March 2017, a 73-year-old female patient was diagnosed with a precursor B-ALL. Flow cytometry showed 95% positivity for CD19 and CD22 surface antigens. First complete remission with no detection of minimal residual disease (MRD) was achieved after a first cycle of standard induction chemotherapy. After six additional cycles of chemotherapy, a molecular relapse occurred followed by an overt relapse in June 2018. The patient was treated with six cycles of InO, inducing a MRD-negative CR without relevant adverse events or alterations in quality of daily life. In March 2019, a second relapse occurred. Lymphocytes were harvested for autologous CAR T cell production within a clinical trial (NCT03853616). While waiting for the CAR T cell product, the patient progressed with symptomatic CNS involvement. Hence, further treatment continued off study and consisted of intrathecal chemotherapy and a further cycle of InO. Subsequently, a partial remission with no evidence of active CNS involvement could be achieved. After a lymphodepleting chemotherapy, the patient received CD19 CAR T cells without any complications or any impairment in quality of life. CAR T cell persistence in the peripheral blood was measurable until day 20 (Fig. 1). Remission control showed a third MRD-negative remission. Five months after CAR T cell therapy, MRD turned positive again, followed by a third overt relapse with involvement of bone marrow and CNS in December 2019. After clearance of blasts in the cerebrospinal fluid by intrathecal chemotherapy, the patient received Blina and achieved a fourth MRD-negative CR after the first cycle. Except mild neurological adverse reactions, treatment was well tolerated and continued for two additional cycles.

Treatment of r/r B-ALL in older patients remains a clinical challenge and is often based on individual decisions. Conventional salvage approaches with intensive chemotherapy (e.g., high-dose AraC ± mitoxantrone, fludarabine/AraC ± idarubicin) are not applicable for most older patients due to high toxicities. Moreover, two recently published randomized trials have shown inferior response rates and survival outcomes compared to novel immunotherapies [4, 6]. Allogeneic stem cell transplantation as consolidation for those patients achieving a CR is of particular importance with regard to long-term outcome. In older patients, this option is only possible in selected fit patients [10]. So far, data on CAR T cell therapy in older patients with B-precursor ALL and active CNS involvement are limited. In general, the observed CAR T cell-related toxicities such as cytokine release syndrome or neurologic toxicities in older patients seemed to be comparable in those seen in younger patients [11]. Our case report demonstrates efficacy and tolerability of CD19-directed CAR T cell therapy in an elderly patient with r/r B-ALL. Given the debulking capacity of InO and the distinct type of targeted antigen makes it rational to apply InO as a bridging therapy prior to CAR T cell application. For CD19+ relapses, Blina can be considered as effective salvage treatment, even after CAR T cell therapy. This case report demonstrates that novel immuno-salvage therapies represent an effective, safe, and feasible treatment option in older patients with r/r B-ALL without impairment of their quality of life. The optimal sequence of novel targeted therapies has to be defined in the future.
